# The relationship between dietary methionine and growth, digestion, absorption, and antioxidant status in intestinal and hepatopancreatic tissues of sub-adult grass carp (*Ctenopharyngodon idella*)

**DOI:** 10.1186/s40104-017-0194-0

**Published:** 2017-08-01

**Authors:** Pei Wu, Ling Tang, Weidan Jiang, Kai Hu, Yang Liu, Jun Jiang, Shengyao Kuang, Ling Tang, Wuneng Tang, Yongan Zhang, Xiaoqiu Zhou, Lin Feng

**Affiliations:** 1 Animal Nutrition Institute, Sichuan Agricultural University, Chengdu, 611130 China; 2 Fish Nutrition and Safety Production University Key Laboratory of Sichuan Province, Sichuan Agricultural University, Chengdu, 611130 China; 3 Key Laboratory for Animal Disease-Resistance Nutrition of China Ministry of Education, Sichuan Agricultural University, Chengdu, 611130 China; 4 Animal Nutrition Institute, Sichuan Academy of Animal Science, Chengdu, 610066 China; 5 Institute of Hydrobiology, Chinese Academy of Sciences, Wuhan, 430072 China

**Keywords:** Antioxidant status, *Ctenopharyngodon idella*, Digestive and absorptive capacities, Methionine

## Abstract

**Background:**

Methionine is an essential amino acid for fish. The present study was conducted to investigate the effects of dietary methionine on growth performance, digestive and absorptive ability, as well as antioxidant capacity in the intestine and hepatopancreas of sub-adult grass carp (*Ctenopharyngodon idella*).

**Results:**

Dietary methionine deficiency significantly decreased percentage weight gain (PWG), feed intake, feed efficiency and protein efficiency ratio, as well as activities of hepatopancreatic glutamate-oxaloacetate transaminase and muscle glutamate-pyruvate transaminase in sub-adult grass carp (*P* < 0.05). Furthermore, methionine deficiency significantly reduced activities of trypsin, lipase and amylase in the intestine, Na^+^/K^+^-ATPase, alkaline phosphatase and γ-glutamyl transpeptidase in three intestinal segments, and creatine kinase (CK) in the proximal intestine (*P* < 0.05). However, an unexplained and significant increase in CK activity in the mid intestine was associated with dietary methionine deficiency. Malondialdehyde and protein carbonyl contents in the intestine and hepatopancreas were significantly increased by methionine deficiency (*P* < 0.05), whereas anti-hydroxyl radical capacity in the hepatopancreas and intestine, and anti-superoxide anion capacity in the intestine, were significantly decreased by methionine deficiency (*P* < 0.05). Moreover, methionine deficiency significantly decreased superoxide dismutase and glutathione reductase activities, glutathione contents in the hepatopancreas and intestine, as well as glutathione peroxidase activity in the intestine (*P* < 0.05), whereas it significantly increased activities of catalase in the hepatopancreas and glutathione-S-transferase in the hepatopancreas and intestine (*P* < 0.05).

**Conclusions:**

The present results demonstrated that dietary methionine deficiency induced poor growth, and decreased digestive and absorptive function and antioxidant capacity in the hepatopancreas and intestine of sub-adult grass carp. Methionine requirements for sub-adult grass carp (450-1, 170 g) based on PWG, intestinal trypsin, and hepatopancreatic anti-hydroxyl radical activities were estimated to be 6.12 g/kg diet (21.80 g/kg protein), 6.99 g/kg diet (24.90 g/kg protein) and 5.42 g/kg diet (19.31 g/kg protein), respectively, in the presence of 1.50 g cysteine/kg (5.35 g/kg protein).

## Background

Methionine (Met) is an essential amino acid for fish [1]. Dietary Met deficiency has been shown to cause poor growth and feed efficiency in juvenile Jian carp (*Cyprinus carpio* var. Jian) [2], fingerling rohu (*Labeo rohita*) [3], juvenile Cobia (*Rachycentron canadum*) [4], juvenile hybrid striped bass (*Morone chrysops × M. saxatilis*) [5], and juvenile European sea bass (*Dicentrarchus labrax*) [6]. Poor feed efficiency may result from inefficient digestion of feed, which depends in part on digestive and absorptive capacities of fish [7]. Fish digestion and absorption abilities depend in turn on the activities of digestive and brush border enzymes, such as trypsin, lipase, amylase, alkaline phosphatase (AKP), creatine kinase (CK), γ-glutamyl transpeptidase (γ-GT), and Na^+^/K^+^-ATPase [8]. To date, there is only one study in omnivorous fish on the relationship between methionine and the brush border enzymes, which showed that methionine improved activities of γ-GT and CK in juvenile Jian carp [2]. However, digestive and brush border enzymes activities may change with feeding habits and growth stage of fish. It has been reported that activities of protease and lipase were generally lower in herbivorous fish species than in omnivorous and carnivores species, whereas amylase activity showed the opposite trend [9]. Meanwhile, the activities of pepsin and trypsin in *Pelteobagrus fulvidraco* larvae decreased with increasing fish age [10]. Moreover, methionine metabolism may vary with growth stage of life-cycle. Nagata et al. [11] reported that the uptake of methionine in the brain of children gradually increased with age. In rat, activity of liver γ-cystathionase, a key enzyme for the transsulfuration of methionine, was lower in newborns than in adults [12]. Therefore, it is worth to investigate the effects of methionine on the activities of digestive and brush border enzymes in sub-adult herbivorous fish.

In fish, digestive function is largely dependent on the growth and development of the intestine and hepatopancreas [13], which is closely related to the structural integrity of tissues. However, oxidative stress that induced by excessive reactive oxygen species (ROS) typically leads to the peroxidation of lipids, and the oxidation of proteins and DNA, resulting in cell damage and organ dysfunction in fish [14]. Methionine supplementation decreased lipid peroxidation in the liver of juvenile hybrid striped bass [5]. Nevertheless, there is no information regarding the effect of Met on protein oxidation and ROS scavenging in fish. In mice, the oxidation/reduction cycle of methionine can destroy ROS [15]. Meanwhile, methionine and its intermediate metabolites, S-adenosylmethionine (SAM) and cysteine, play a role in chelating Fe^2+^ and Cu^+^, consequently decreasing OH˙ formation in vitro [16, 17]. These observations suggest that Met might play a role in scavenging ROS in fish, which warrants investigation.

As in terrestrial animals, antioxidant enzymes and non-enzymatic compounds play key roles in scavenging ROS in fish [14]. To date, no study has investigated the relationship between Met and antioxidant system in the digestive organs of fish. A few studies reported that methionine increased liver glutathione (GSH) content in juvenile sunshine bass (*Morone chrysops*♀× *M. saxatilis*♂) [18], and activities of superoxide dismutase (SOD), catalase (CAT), glutathione peroxidase (GPx) in gilthead sea bream (*Sparus aurata*) [19, 20]. Furthermore, an earlier study from our laboratory has shown that methionine hydroxy analogue (MHA), which can be converted into L-methionine in chicken small intestine [21], enhanced GSH content and activities of antioxidant enzymes, including SOD, CAT, GPx, glutathione-S-transferase (GST) and glutathione reductase (GR) in the intestine and hepatopancreas of juvenile Jian carp [22]. Accordingly, methionine might affect enzymatic antioxidant capacity and non-enzymatic compounds in fish digestive organs; however, these relationships remain to be characterization.

The grass carp (*Ctenopharyngodon idella*) is a commercially important herbivorous species with a global distribution [23]. Grass carp culturing relies heavily on the use of plant feedstuffs, which are known to contain low levels of methionine [24]. It has been reported that the methionine requirement for juvenile grass carp based on weight gain was 11 g/kg diet (29.7 g/kg protein) [25]. However, nutrient requirements might vary with fish growth stages. The requirement of methionine for max growth of juvenile common carp was higher than that for adult common carp [26, 27]. Similarly, the protein requirement for max growth of grass carp decreased with increasing fish size [23, 28]. Hence, it is valuable to evaluate the methionine requirements of grass carp at the sub-adult growth stage.

Therefore, the aim of the present study was to investigate the effects of dietary methionine on growth performance, digestive and absorptive ability, and antioxidant capacity in the intestine and hepatopancreas of sub-adult grass carp. In addition, dietary methionine requirements for sub-adult grass carp were estimated.

## Methods

### Experimental design and diets

Fishmeal, casein, gelatin and crystalline amino acid served as dietary protein sources (Table 1). Fish oil and soybean oil were used as dietary lipid sources. Apart from Met, The dietary amino acid profile was similar to that of whole chicken egg protein according to Abidi and Khan [3]. The six experimental diets were kept isonitrogenous by decreasing L-glycine levels as methionine levels increased. Dietary protein and lipid were determined to be 280.6 g/kg diet and 42.2 g/kg diet, respectively, according to the standard methods of AOAC [29]. The basal diet contained 1.5 g cysteine/kg diet, and the methionine concentrations of the six experimental diets were 2.21 (unsupplemented control group), 4.24, 6.22, 8.25, 10.24 and 12.26 g/kg diet, as determined by reverse-phase high performance liquid chromatography (HPLC, HP 1100, USA). The diets were prepared according to the method described by Mai et al. [30]. In brief, ingredients were ground into a fine powder through a 300 μm screen. Oil and water were added to the premixed dry ingredients and thoroughly mixed until homogenous. The wet dough was adjusted to pH 7.0 using 6.0 mol/L NaOH according to the method proposed by Zhou et al. [4], then extruded through a mincer with die and fan-dried at room temperature. The diets were then broken up and sieved into pellets (3.5 mm × 5.0 mm), and stored at −20 °C according to the method described by Quintero et al. [31].

**Table 1:** Composition of the experimental diets

	Diets, g/kg					
Ingredients	Diet 1	Diet 2	Diet 3	Diet 4	Diet 5	Diet 6
Anchovy fish meal^a^	68.00	68.00	68.00	68.00	68.00	68.00
Casein^a^	30.00	30.00	30.00	30.00	30.00	30.00
Gelatin^a^	39.90	39.90	39.90	39.90	39.90	39.90
Amino acid mix^b^	150.22	150.22	150.22	150.22	150.22	150.22
α-Starch	280.00	280.00	280.00	280.00	280.00	280.00
Anchovy fish oil^c^	22.80	22.80	22.80	22.80	22.80	22.80
Soybean oil	18.90	18.90	18.90	18.90	18.90	18.90
Vitamin premix^d^	10.00	10.00	10.00	10.00	10.00	10.00
Mineral premix^e^	20.00	20.00	20.00	20.00	20.00	20.00
Ca(H_2_PO_4_)_2_	22.90	22.90	22.90	22.90	22.90	22.90
Choline chloride (500 g/kg)	6.00	6.00	6.00	6.00	6.00	6.00
α-Cellulose	150.00	150.00	150.00	150.00	150.00	150.00
Ethoxyquin (300 g/kg)	0.50	0.50	0.50	0.50	0.50	0.50
DL-Methionine	0.00	2.02	4.04	6.06	8.08	10.10
Glycine	49.09	48.08	47.07	46.06	45.05	44.04
Corn starch	131.69	130.68	129.67	128.66	127.65	126.64

^a^Fish meal was purchased from Pesquera Lota Protein Ltd. (Lota, Chile), casein was purchased from Hulunbeier Sanyuan Milk Co., Ltd. (Inner Mongolia, China), gelatin was purchased from Rousselot Gelatin Co., Ltd. (Guangdong, China)

^b^Amino acid mix (g/kg): arginine (100%), 11.80 g; histidine (73.3%), 7.23 g; isoleucine (99%), 11.82 g; leucine (99%), 18.99 g; lysine (78.8%), 15.99 g; cystine (99%), 0.81 g; phenylalanine (99%), 12.53 g; tyrosine (99%), 10.00 g; threonine (98%), 11.22 g; tryptophan (98%), 3.27 g; valine (99%), 14.24 g; glutamic acid (99%), 32.32 g

^c^Fish oil was purchased form CIA. Pesquera Camanchaca S.A. (Santiago, Chile)

^d^Per kilogram of vitamin premix (g/kg): retinyl acetate (500,000 IU/g), 0.800 g; cholecalciferol (500,000 IU/g), 0.480 g; DL-α-tocopherol acetate (500 g/kg), 20.000 g; menadione (23%), 0.220 g; thiamine hydrochloride (98%), 0.120 g; riboflavin (80%), 0.990 g; pyridoxine hydrochloride (98%), 0.620 g; cyanocobalamin (1%), 0.100 g; niacin (99%), 2.58 g; D-biotin (2%), 5.000 g; meso-inositol (99%), 52.330 g; folic acid (96%), 0.520 g; ascorhyl acetate (93%), 7.160 g; calcium-D-pantothenate (90%), 2.780 g. All ingredients were diluted with corn starch to 1 kg

^e^Per kilogram of mineral premix (g/kg): FeSO_4_·H_2_O, 25.00 g; CuSO_4_·5H_2_O, 0.60 g; ZnSO_4_·H_2_O, 4.35 g; MnSO_4_·H_2_O, 2.04 g; KI, 1.10 g; NaSeO_3_, 2.50 g; MgSO_4_·H_2_O, 230.67 g. All ingredients were diluted with corn starch to 1 kg

### Fish management and feeding

All procedures used in this study were approved by the Institutional Animal Care and Use Committee of Sichuan Agricultural University. Grass carp were obtained from a commercial farm in Bailong Lake, Sichuan, China. Fish were reared in cages (1.4 m × 1.4 m × 1.4 m) settled in culture ponds for 2 wk. prior to the experiment to adapt to the experimental environment, and were fed a commercial diet that represented the baseline nutrient source of grass carp. A total of 600 grass carps with an average initial weight of 451.3 ± 1.1 g were randomly distributed into 30 experimental cages at a density of 20 fish per cage. Each of the experimental diets was randomly assigned to five cages. Each cage was equipped with a disc of 80 cm diameter and 1-mm gauze in the bottom to collect the uneaten food, as described by Sveier et al. [32], with minor modification. In the present study, the uneaten feed was removed by siphoning after 30 min of feeding [33] and was dried and weighed. The actual amount of feed consumed was calculated on a dry-matter basis according to the method described by Helland et al. [34]. Water, from a river was pumped continuously through sand filters and flowed into each cage at the rate of 1 L/min to remove impurities and reduce the ammonia concentrations according to the methods of Chen et al. [35]. Water quality parameters were measured daily using a YSI Professional Plus Multiparameter Instrument (YSI Incorporated, Yellow Springs, OH, USA). Water temperature and pH were 25 ± 2 °C and 7.5 ± 0.3 respectively, and dissolved oxygen was maintained at concentration higher than 6.0 mg/L by connecting each cage to an oxygen auto-supplemention system employing micropore aeration. The fish were fed the assigned diet four times daily until apparent satiation for 8 wk.

### Sample collection and analysis

Fish in each cage were counted and weighted at the beginning and end of the 8-week feeding test. Twelve h after the last feeding, 15 fish from each replicate were anaesthetised in benzocaine bath (50 mg/L) as described by Berdikova Bohne et al. [36]. The whole intestine, hepatopancreas and muscle were quickly removed, weighed, frozen in liquid nitrogen, and stored at −70 °C until analysis.

Intestine, hepatopancreas and muscle tissue samples were homogenized on ice in 10 volumes (*w*/*v*) ice-cold physiological saline and centrifuged at 6000 × *g* for 20 min at 4 °C. The supernatant was collected and stored at −70 °C for enzyme activity analysis. Trypsin activity was measured according to the method described by Hummel [37], lipase and amylase activities were assayed according to Furné et al. [38], and activities of Na^+^/K^+^-ATPase, AKP, γ-GT and CK were measured according to McCormick [39], Bessey [40], Bauermeister et al. [41] and Weng et al. [42], respectively. Activities of glutamic-oxaloacetic transaminase (GOT) and glutamate-pyruvic transaminase (GPT) were measured by the methods of Bergmeyer and Bernt [43, 44]. Malondialdehyde (MDA) and protein carbonyl (PC) contents were assayed according to Zhang et al. [45]. The capacities of anti-superoxide anion (ASA) (O_2_^–^·-scavenging ability) and anti-hydroxy radical (AHR) (·OH-scavenging ability) were measured using the methods described by Jiang et al. [46]. The activities of SOD and GPx were measured by the method of Zhang et al. [45], and activities of CAT, GST and GR were measured as described by Aebi [47], Lushchak et al. [48] and Lora et al. [49], respectively. GSH content was measured according to Vardi et al. [50]. Protein content was assayed by the method of Bradford [51].

### Calculation and statistical analysis

The following variables were calculated:

Survival rate (SR, %) = final amount of fish/initial amount of fish × 100.

Percent weight gain (PWG, %) = (final body weight - initial body weight)/initial body weight ×100.

Feed intake (FI, g/fish) = (feed offered in dry basis - uneaten feed in dry basis/recovery of uneaten feed in dry basis)/amount of fish [34].

Feed efficiency (FE, %) = weight gain (g)/feed intake in dry basis (g) × 100.

Protein efficiency ratio (PER) = weight gain (g)/protein intake (g).

Hepatosomatic index (HSI, %) = wet hepatopancreas weight (g)/wet body weight (g) × 100.

Intestosomatic index (ISI, %) = wet intestine weight (g)/wet body weight (g) × 100.

Hepatopancreas protein content (HPC, %) = hepatopancreas protein (g)/wet hepatopancreas weight (g) × 100.

Intestinal protein content (IPC, %) = intestine protein (g)/wet intestine weight (g) × 100.

Relative gut length (RGL, %) = intestine length (cm)/total body length (cm) × 100.

Results were expressed as means ± SD. All data were subjected to one-way analysis of variance (ANOVA) followed by Duncan’s multiple-range test to determine significant differences among treatments at the level of *P* < 0.05 through SPSS 13.0 (SPSS Inc., Chicago, IL, USA). The relationship between dietary methionine and growth performance, activities of digestive and brush border enzymes, as well as antioxidant enzymes in the hepatopancreas and intestine were respectively subjected to a linear regression or quadratic regression model. For each variable, the regression analysis that gave the least mean square error was considered the best fitted model, and was used to estimate the dietary Met requirements according to the method of Zeitoun et al. [52].

## Results

### Growth performance

**Fig. 1:**
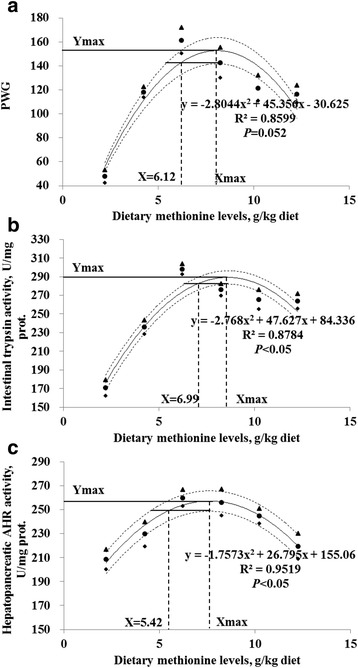
Quadratic regression analysis (solid curved lines) of percentage weight gain (PWG), intestinal trypsin and hepatopancreatic anti-hydroxy radical (AHR) activities for sub-adult grass carp, with 0.95 confidence limits (dashed curved lines). ▲ and ◆ Estimated mean of Y at 0.95 confidence limits; ● Observed mean of Y. **a** PWG; **b** Intestinal trypsin activity; **c** Hepatopancreatic AHR activity

No mortality was observed during the experiment. As shown in Table 2, percentage weight gain (PWG) and feed intake (FI) were significantly increased with increasing levels of dietary methionine up to 6.22 g/kg diet, and declined significantly thereafter. Feed efficiency (FE) and protein efficiency ratio (PER) increased significantly with increasing levels of dietary methionine from 2.21 g/kg diet to 4.24 g/kg diet, and then plateaued. Regression analysis showed that PWG and FI were quadratic responses to increasing dietary methionine levels (Table 2). The methionine requirement of grass carp (450-1, 170 g), established by quadratic regression analysis based on PWG, was 6.12 g/kg diet (21.80 g/kg protein) in the presence of 1.50 g cysteine/kg diet (5.35 g/kg protein) (Fig. 1a).

**Table 2:** Initial body weight (IBW), percentage weight gain (PWG), food intake (FI), feed efficiency (FE), and protein efficiency ratio (PER) of sub-adult grass carp fed diets with graded levels of methionine for 8 wk^1^

Met, g/kg diet	2.21	4.24	6.22	8.25	10.24	12.26
IBW, g/fish	450.6 ± 0.89	452 ± 1.58	451.4 ± 0.55	450.8 ± 0.84	451 ± 1.00	452 ± 1.00
PWG, %	47.53 ± 4.23^a^	117.87 ± 3.43^b^	161.10 ± 8.58^d^	142.60 ± 10.09^c^	121.17 ± 8.30^b^	116.28 ± 5.69^b^
FI, g/fish	522.3 ± 4.6^a^	952.5 ± 1.4^c^	1249.8 ± 27.8^f^	1164.9 ± 6.9^e^	996.7 ± 4.0^d^	905.8 ± 1.2^b^
FE, %	46.04 ± 3.82^a^	62.84 ± 1.93^b^	65.38 ± 3.48^b^	61.99 ± 4.33^b^	61.60 ± 4.40^b^	65.18 ± 3.24^b^
PER	1.46 ± 0.12^a^	2.00 ± 0.06^b^	2.08 ± 0.11^b^	1.97 ± 0.14^b^	1.96 ± 0.14^b^	2.07 ± 0.10^b^
Regressions						
Y_PWG_ = −2.805X^2^ + 45.358X – 30.628				*R* ^2^ = 0.860		*P* = 0.052
Y_FI_ = −19.734X^2^ + 313.563X – 38.287				*R* ^2^ = 0.909		*P* = 0.013

^a-f^Means in the same row with different superscript letters differ significantly (*P* < 0.05)

^1^Values are mean ± SD (*n* = 5). Survival among all dietary treatments was 100%

### Activities of GOT and GPT in the hepatopancreas and muscle

GOT activity in the hepatopancreas was significantly increased with increasing levels of dietary methionine up to 10.24 g/kg diet, and declined significantly thereafter (Table 3). GPT activities in the muscle of groups fed diets containing 6.22 and 8.25 g/kg methionine were significantly higher than those of all other groups. However, activities of GPT in hepatopancreas and GOT in muscle showed no significant difference among groups. Regression analysis showed that activity of GPT in the muscle quadratically responded to increased dietary methionine levels (Table 3).

**Table 3:** Activities of glutamate-oxaloacetate transaminase (GOT) and glutamate-pyruvate transaminase (GPT) in the hepatopancreas and muscle of sub-adult grass carp fed diets with graded levels of methionine for 8 wk^1^

Met, g/kg diet	2.21	4.24	6.22	8.25	10.24	12.26
Hepatopancreas						
GOT, U/g protein	13.82 ± 1.50^a^	22.83 ± 1.81^c^	23.88 ± 1.66^c^	24.35 ± 1.12^c^	32.43 ± 2.01^d^	20.97 ± 1.09^b^
GPT, U/g protein	21.30 ± 1.44	21.54 ± 1.79	20.97 ± 0.96	20.60 ± 0.88	21.16 ± 1.24	20.60 ± 1.26
Muscle						
GOT, U/g protein	15.15 ± 1.37	15.87 ± 1.47	15.95 ± 1.35	15.96 ± 1.33	15.41 ± 0.76	15.11 ± 1.41
GPT, U/g protein	3.46 ± 0.26^b^	4.64 ± 0.34^c^	10.74 ± 0.59^e^	11.25 ± 0.58^e^	6.92 ± 0.57^d^	1.45 ± 0.12^a^
Regression						
Y_GPT in muscle_ = −0.331X^2^ + 4.747X – 6.734				*R* ^2^ = 0.846		*P* = 0.060

^a-e^Means in the same row with different superscript letters differ significantly (*P* < 0.05)

^1^Values are mean ± SD (*n* = 6)

### Activities of digestive and brush border enzymes in the intestine

The digestive and brush border enzyme activities in the intestine were significantly affected by dietary methionine levels (Table 4). Activity of trypsin was significantly increased with increasing levels of dietary methionine up to 6.22 g/kg diet and declined significantly thereafter, following a quadratic relationship (Table 4). Lipase activity in the group fed 6.22 g methionine/kg diet was significantly higher than those in other groups, and among which there were no significant differences. Amylase activity in the group fed 2.21 g methionine/kg diet was significantly lower than in the other dietary treatments. Na^+^/K^+^-ATPase activities in the proximal intestine (PI), mid intestine (MI) and distal intestine (DI) increased significantly with increase of dietary methionine levels up to 10.24, 8.25 and 6.22 g/kg diet, respectively, and then declined significantly. AKP activities for PI at methionine level of 6.22 g/kg and DI at methionine level of 8.25 g/kg were significantly higher than those for the other treatments. The activity of AKP in the MI was significantly increased with increasing dietary methionine levels up to 8.25 g/kg diet, and then plateaued. γ-GT activities in the PI and MI were increased significantly with increasing dietary methionine levels up to 4.24 and 6.22 g/kg diet, respectively, and declined significantly with further increase of dietary methionine. γ-GT activity in the DI was significantly increased with increase of methionine levels up to 6.22 g/kg diet, and plateaued thereafter. CK activity in the PI was significantly improved with dietary methionine levels up to 6.22 g/kg diet. However, in the MI, CK activity decreased significantly with increasing dietary methionine levels up to 10.24 g/kg diet, whereas, in the DI, there was showed no significant difference among groups. Regression analysis indicated that the activities of Na^+^/K^+^-ATPase in MI, γ-GT in DI, AKP and CK in PI and MI quadratically responded to increasing dietary methionine levels (Table 4). Based on the quadratic regression analysis of intestinal trypsin activity, the methionine requirement of grass carp (450-1, 170 g) was estimated to be 6.99 g/kg diet (24.90 g/kg protein) in the presence of 1.50 g cysteine/kg diet (5.35 g/kg protein) (Fig. 1b).

**Table 4:** Effects of methionine on the activities of trypsin, lipase and amylase in the intestine, Na^+^/K^+^-ATPase, alkaline phosphatase (AKP), γ-glutamyl transpeptidase (γ-GT) and creatine kinase (CK) in the proximal intestine (PI), mid intestine (MI) and distal intestine (DI) of sub-adult grass carp^1^

Met, g/kg diet	2.21	4.24	6.22	8.25	10.24	12.26
Trypsin, U/mg protein	170.42 ± 8.13^a^	235.75 ± 7.31^b^	298.06 ± 5.53^e^	275.83 ± 5.93^d^	265.32 ± 9.89^c^	263.54 ± 7.52^c^
Lipase, U/g protein	26.92 ± 1.58^a^	28.54 ± 2.21^a^	44.78 ± 4.82^b^	28.54 ± 2.59^a^	29.31 ± 3.12^a^	27.24 ± 2.28^a^
Amylase, U/mg protein	1.78 ± 0.09^a^	1.95 ± 0.08^b^	1.90 ± 0.06^b^	1.88 ± 0.09^b^	1.90 ± 0.03^b^	1.94 ± 0.04^b^
Na^+^/K^+^-ATPase, μmol pi/mg protein per hour						
PI	0.56 ± 0.03^ab^	0.56 ± 0.04^ab^	0.66 ± 0.06^c^	0.62 ± 0.04^bc^	0.74 ± 0.09^d^	0.51 ± 0.02^a^
MI	0.52 ± 0.04^bc^	0.55 ± 0.05^bcd^	0.56 ± 0.04^cd^	0.59 ± 0.05^d^	0.50 ± 0.04^b^	0.44 ± 0.03^a^
DI	0.41 ± 0.03^a^	0.43 ± 0.04^ab^	0.47 ± 0.05^b^	0.40 ± 0.04^a^	0.40 ± 0.04^a^	0.39 ± 0.04^a^
AKP, U/g protein						
PI	95.50 ± 10.36^a^	114.14 ± 8.49^b^	187.58 ± 20.75^e^	167.47 ± 13.52^d^	167.87 ± 14.96^d^	133.74 ± 13.56^c^
MI	37.84 ± 4.02^a^	51.76 ± 5.44^b^	52.45 ± 4.17^b^	75.72 ± 7.24^c^	71.65 ± 7.42^c^	72.29 ± 8.36^c^
DI	43.89 ± 2.68^a^	46.89 ± 2.70^ab^	48.87 ± 2.51^bc^	59.24 ± 3.37^d^	51.74 ± 3.38^c^	44.72 ± 3.75^a^
γ-GT, U/g protein						
PI	3.62 ± 0.18^a^	8.55 ± 0.28^e^	6.83 ± 0.36^d^	5.89 ± 0.36^c^	5.92 ± 0.24^c^	4.92 ± 0.18^b^
MI	5.01 ± 0.39^a^	7.01 ± 0.22^c^	7.07 ± 0.40^c^	5.62 ± 0.32^b^	5.56 ± 0.22^b^	4.79 ± 0.16^a^
DI	0.52 ± 0.04^a^	1.69 ± 0.14^b^	2.07 ± 0.13^c^	2.21 ± 0.17^c^	2.19 ± 0.13^c^	2.18 ± 0.16^c^
CK, U/mg protein						
PI	1.91 ± 0.21^a^	2.28 ± 0.19^b^	3.75 ± 0.25^d^	3.63 ± 0.37^cd^	3.41 ± 0.37^c^	2.17 ± 0.22^ab^
MI	1.65 ± 0.13^e^	1.46 ± 0.14^d^	0.91 ± 0.09^c^	0.71 ± 0.05^b^	0.54 ± 0.04^a^	0.59 ± 0.07^a^
DI	3.43 ± 0.18	3.36 ± 0.27	3.37 ± 0.17	3.24 ± 0.36	3.29 ± 0.34	3.27 ± 0.26
Regressions						
Y _Trypsin_ = −2.768X^2^ + 47.626X + 84.338				*R* ^2^ = 0.878		*P* = 0.042
Y _Na_^+^_/K_^+^_-ATPase in MI_ = −0.004X^2^ + 0.047X + 0.428				*R* ^2^ = 0.893		*P* = 0.035
Y _AKP in PI_ = −2.452X^2^ + 40.210X + 10.672				*R* ^2^ = 0.822		*P* = 0.075
Y _AKP in MI_ = −0.378X^2^ + 9.100X + 18.654				*R* ^2^ = 0.886		*P* = 0.038
Y_γ-GT in DI_ = −0.033X^2^ + 0.622X – 0.561				*R* ^2^ = 0.953		*P* = 0.010
Y_CK in PI_ = −0.065X^2^ + 1.011X – 0.264				*R* ^2^ = 0.852		*P* = 0.057
Y_CK in MI_ = 0.012X^2^–0.291X + 2.313				*R* ^2^ = 0.962		*P* = 0.007

^a-e^Means in the same row with different superscript letters differ significantly (*P* < 0.05)

^1^Values are mean ± SD (*n* = 6)

### Hepatopancreas and intestinal growth

There were no differences in hepatosomatic index (HSI) between fish in the group fed 2.21 g methionine/kg diet and those in the other five groups (Table 5). However, HSI in groups fed methionine at levels of 4.24 and 12.26 g/kg diet was significantly higher than those in groups fed with 8.25 and 10.24 g methionine/kg diet. Hepatopancreas protein content (HPC) increased significantly with increasing dietary methionine levels at 6.22 g/kg diet, and no significant differences were found with further increase of methionine. Intestosomatic index (ISI) increased significantly with increasing methionine levels at 4.24 g/kg diet, and declined significantly thereafter. No differences were found for relative gut length (RGL) and intestine protein content (IPC) among dietary treatments (Table 5).

**Table 5:** The hepatosomatic index (HSI), intestosomatic index (ISI), relative gut length (RGL), hepatopancreas protein content (HPC) and intestinal protein content (IPC) of sub-adult grass carp fed with graded levels of methionine

Met, g/kg diet	2.21	4.24	6.22	8.25	10.24	12.26
HSI^1^, %	2.03 ± 0.32^ab^	2.17 ± 0.35^b^	1.98 ± 0.29^ab^	1.85 ± 0.32^a^	1.91 ± 0.29^a^	2.19 ± 0.28^b^
HPC^2^, %	8.74 ± 0.43^a^	8.74 ± 0.33^a^	9.84 ± 0.55^b^	9.66 ± 0.43^b^	9.40 ± 0.68^ab^	9.40 ± 0.72^ab^
ISI^1^, %	1.27 ± 0.19^b^	1.39 ± 0.16^c^	1.24 ± 0.15^b^	1.07 ± 0.13^a^	1.06 ± 0.11^a^	1.16 ± 0.18^ab^
RGL^1^, %	148.7 ± 16.25	148.7 ± 8.97	150.6 ± 12.61	146.2 ± 12.56	142.0 ± 14.39	143.7 ± 9.26
IPC^2^, %	9.88 ± 0.92	9.65 ± 1.02	10.19 ± 1.05	9.79 ± 0.63	9.65 ± 1.02	9.38 ± 0.83

^a-c^Means in the same row with different superscript letters differ significantly (*P* < 0.05)

^1^Values are mean ± SD (*n* = 15)

^2^Values are mean ± SD (*n* = 6)

### Antioxidant indicators in hepatopancreas and intestine

MDA content in the hepatopancreas was significantly lower in fish fed 6.22 g methionine/kg diet compared with the other groups, and showed no significant difference among other groups (Table 6). Hepatopancreatic PC content was decreased with an increase in the dietary methionine levels up to 6.22 g/kg diet, and then increased significantly. In the intestine, MDA content was declined with increasing methionine levels up to 6.22 and 8.25 g/kg diet, and enhanced significantly thereafter. PC content in the intestine decreased significantly with increasing dietary methionine levels up to 4.24 g/kg diet, and remained a plateau with incremental dietary methionine levels from 4.24 to 10.24 g/kg diet, then increased significantly. AHR capacity in the hepatopancreas was significantly improved by increased levels of dietary methionine up to 6.22 and 8.25 g/kg diet, and then declined significantly, whereas ASA capacity was not affected significantly by dietary methionine levels. In the intestine, the AHR capacity significantly increased with increase of methionine levels up to 6.22 g/kg diet, and then decreased significantly. ASA capacity was increased with increasing methionine levels up to 4.24 g/kg diet, and then plateaued. Regression analysis showed that MDA content and ASA in the intestine, as well as PC and AHR in the hepatopancreas and intestine were quadratic responses to dietary methionine levels (Table 6). The methionine requirement of grass carp (450-1, 170 g), determined by quadratic regression analysis based on hepatopancreatic AHR activity, was 5.42 g/kg diet (19.31 g/kg protein) in the presence of 1.50 g cysteine/kg diet (5.35 g/kg protein) (Fig. 1c).

**Table 6:** Effects of methionine on malondialdehyde (MDA), protein carbonyl (PC) content, anti-superoxide anion (ASA) and anti-hydroxy radical (AHR) activities in the hepatopancreas and intestine of sub-adult grass carp^1^

Met, g/kg diet	2.21	4.24	6.22	8.25	10.24	12.26
Hepatopancreas						
MDA, nmol/mg protein	2.19 ± 0.18^b^	2.09 ± 0.10^b^	1.15 ± 0.11^a^	2.04 ± 0.14^b^	2.15 ± 0.15^b^	2.08 ± 0.08^b^
PC, nmol/mg protein	6.63 ± 0.48^c^	3.66 ± 0.31^b^	2.56 ± 0.20^a^	3.53 ± 0.36^b^	3.67 ± 0.29^b^	3.63 ± 0.14^b^
ASA, U/g protein	197.0 ± 15.3	194.4 ± 8.7	202.9 ± 12.9	205.6 ± 15.2	202.9 ± 16.80	202.5 ± 7.3
AHR, U/mg protein	208.5 ± 8.0^a^	229.5 ± 9.7^c^	259.7 ± 6.4^e^	256.0 ± 10.6^e^	244.7 ± 5.90^d^	219.3 ± 10.1^b^
Intestine						
MDA, nmol/mg protein	1.73 ± 0.14^b^	1.72 ± 0.17^b^	1.44 ± 0.08^a^	1.41 ± 0.11^a^	1.98 ± 0.12^c^	2.09 ± 0.12^c^
PC, nmol/mg protein	4.27 ± 0.34^b^	3.63 ± 0.36^a^	3.16 ± 0.38^a^	3.19 ± 0.39^a^	3.19 ± 0.26^a^	5.30 ± 0.37^c^
ASA, U/g protein	213.5 ± 19.0^a^	281.6 ± 19.4^b^	303.0 ± 24.5^b^	284.9 ± 20.7^b^	282.1 ± 25.2^b^	287.3 ± 23.5^b^
AHR, U/mg protein	133.4 ± 8.7^a^	173.2 ± 13.0^b^	198.1 ± 12.8^c^	179.6 ± 11.9^b^	175.8 ± 12.8^b^	143.2 ± 12.7^a^
Regressions						
Y _PC in Hepatopancreas_ = 0.087X^2^–1.455X + 8.910				*R* ^2^ = 0.773		*P* = 0.108
Y _AHR in Hepatopancreas_ = −1.757X^2^ + 26.793X + 155.081				*R* ^2^ = 0.952		*P* = 0.010
Y _MDA in Intestine_ = 0.018X^2^–0.219X + 2.181				*R* ^2^ = 0.745		*P* = 0.129
Y _PC in Intestine_ = 0.069X^2^–0.945X + 6.198				*R* ^2^ = 0.855		*P* = 0.055
Y _ASA in Intestine_ = −1.819X^2^ + 31.349X + 165.218				*R* ^2^ = 0.773		*P* = 0.108
Y _AHR in Intestine_ = −2.108X^2^ + 31.047X + 77.708				*R* ^2^ = 0.929		*P* = 0.019

^a-e^Means in the same row with different superscript letters differ significantly (*P* < 0.05)

^1^Values are mean ± SD (*n* = 6)

Activity of SOD in the hepatopancreas was significantly enhanced by increased dietary methionine levels up to 6.22 g/kg diet, and declined thereafter (Table 7). Meanwhile, GR activity and GSH content in the hepatopancreas showed a trend similar to that of SOD activity, and increased significantly with increasing methionine levels up to 4.24 and 6.22 g/kg diet. However, the activities of CAT and GST in thehepatopancreas were significantly decreased with increasing dietary methionine levels up to 6.22 and 8.25 g/kg diet, respectively, and showed quadratic responses to dietary methionine levels. GPx activity in the hepatopancreas showed no significant difference among groups. In the intestine, the activities of SOD and GR, as well as content of GSH were significantly enhanced by increasing dietary methionine levels up to 6.22 g/kg diet, and decreased significantly with further increase of methionine levels. GPx activity in the intestine showed a trend similar to that of the SOD activity, and was enhanced significantly with increase of methionine levels up to 4.24 and 6.22 g/kg diet. However, the activity of intestinal GST was decreased significantly by increasing dietary methionine levels up to 6.22 g/kg diet, and then plateaued. Dietary methionine had no significant effect on intestinal CAT activity. Finally, the methionine requirement for grass carp (450-1, 170 g) ranged from 4.51 g/kg diet (16.07 g/kg protein) to 7.11 g/kg diet (25.35 g/kg protein) in the presence of 1.5 g cysteine/kg diet (5.35 g/kg protein) based on different parameters (Table 8).

**Table 7:** Effects of methionine on superoxide dismutase (SOD), catalase (CAT), glutathione-S-transferase (GST), glutathione peroxidase (GPx), glutathione reducase (GR) activities and glutathione (GSH) contents in the hepatopancreas and intestine of sub-adult grass carp ^1^

Met, g/kg diet	2.21	4.24	6.22	8.25	10.24	12.26
Hepatopancreas						
SOD, U/mg protein	85.06 ± 6.35^a^	96.33 ± 8.17^b^	109.44 ± 4.24^c^	97.08 ± 4.47^b^	95.10 ± 6.64^b^	94.63 ± 5.13^b^
CAT, U/mg protein	29.91 ± 2.75^bc^	28.57 ± 2.40^b^	22.03 ± 2.23^a^	27.32 ± 0.97^b^	32.08 ± 3.44^c^	36.53 ± 3.03^d^
GST, U/mg protein	57.57 ± 2.39^d^	28.52 ± 2.45^b^	23.22 ± 2.31^a^	20.79 ± 1.50^a^	26.27 ± 1.72^b^	44.93 ± 2.03^c^
GPx, U/mg protein	1123.5 ± 67.1	1126.8 ± 53.8	1152.7 ± 62.8	1168.2 ± 39.4	1168.0 ± 57.8	1149.5 ± 56.6
GR, U/g protein	3.93 ± 0.35^a^	7.73 ± 0.43^c^	8.21 ± 0.72^c^	4.50 ± 0.41^ab^	4.68 ± 0.38^b^	4.04 ± 0.32^ab^
GSH, mg/g protein	7.11 ± 0.40^ab^	9.11 ± 0.53^c^	8.77 ± 0.61^c^	7.12 ± 0.32^ab^	7.28 ± 0.32^b^	6.60 ± 0.26^a^
Intestine						
SOD, U/mg protein	21.32 ± 1.65^a^	25.52 ± 1.27^b^	29.83 ± 2.41^c^	26.13 ± 2.01^b^	25.65 ± 1.20^b^	25.75 ± 3.03^b^
CAT, U/mg protein	12.80 ± 1.35	14.02 ± 0.93	13.27 ± 0.37	13.48 ± 1.03	12.62 ± 1.2	13.28 ± 1.18
GST, U/mg protein	14.80 ± 0.85^b^	14.88 ± 1.30^b^	12.84 ± 1.26^a^	14.08 ± 1.20^ab^	13.10 ± 1.28^a^	13.94 ± 1.37^ab^
GPx, U/mg protein	85.41 ± 8.12^a^	107.28 ± 11.02^b^	101.91 ± 10.68^b^	89.68 ± 9.60^a^	80.54 ± 6.69^a^	80.98 ± 5.46^a^
GR, U/g protein	15.47 ± 1.36^a^	23.81 ± 2.29^b^	40.44 ± 3.90^c^	26.51 ± 2.28^b^	25.67 ± 2.39^b^	26.27 ± 1.62^b^
GSH, mg/g protein	0.63 ± 0.07^a^	2.82 ± 0.16^e^	3.70 ± 0.24^f^	2.51 ± 0.11^d^	2.31 ± 0.10^c^	2.01 ± 0.14^b^
Regressions						
Y _CAT in Hepatopancreas_ = 0.327X^2^–4.037X + 37.644				*R* ^2^ = 0.846		*P* = 0.061
Y _GST in Hepatopancreas_ = 1.246X^2^–19.063X + 91.601				*R* ^2^ = 0.969		*P* = 0.006

^a-f^Means in the same row with different superscript letters differ significantly (*P* < 0.05)

^1^Values are mean ± SD (*n* = 6)

**Table 8:** Estimated dietary methionine requirements for sub-adult grass carp

Fish weight, g	Response variables	Model	Estimated Met requirements (0.95 confidence limits)
Sub-adult grass carp 450-1, 170	Feed intake	Quadratic regression	7.04 g/kg diet, 25.09 g/kg protein
	Muscle GPT activity	Quadratic regression	5.79 g/kg diet, 20.63 g/kg protein
	AKP activity in PI	Quadratic regression	5.59 g/kg diet, 19.92 g/kg protein
	AKP activity in MI	Linear regression	7.11 g/kg diet, 25.35 g/kg protein
	γ-GT activity in DI	Linear regression	5.73 g/kg diet, 20.43 g/kg protein
	CK activity in PI	Quadratic regression	5.47 g/kg diet, 19.49 g/kg protein
	Hepatopancreatic GST activity	Quadratic regression	6.39 g/kg diet, 22.77 g/kg protein
	Intestinal PC content	Quadratic regression	4.51 g/kg diet, 16.07 g/kg protein
	Intestinal AHR activity	Quadratic regression	4.83 g/kg diet, 17.21 g/kg protein

*GPT* glutamate-pyruvate transaminase, *AKP* alkaline phosphatase, *γ-GT* γ-glutamyl transpeptidase, *CK* creatine kinase, *PC* protein carbonyl, *AHR* anti-hydroxy radical, *GST* glutathione-S-transferase, *PI* proximal intestine, *MI* mid intestine, *DI* distal intestine

## Discussion

The present study showed that sub-adult grass carp fed the methionine-deficient diet developed poor growth. PWG, FI and FE were lower in fish fed the methionine-deficient diet; however, these parameters increased with increasing dietary methionine to an optimal level. Similar trends have been reported in juvenile cobia [4], common carp [27], large yellow croaker (*Pseudosciaena crocea* R) [53], fingerling rohu [3], Atlantic salmon [54], juvenile hybrid striped bass [5], and juvenile Jian carp [2]. Fish growth is positively associated with the accretion of protein, fat, and other nutrients [9]. In the present study, PER was improved by dietary methionine, which is in agreement with the result in juvenile cobia [4]. Correlation analysis showed that PWG was positively correlated to PER (r = + 0.901, *P* < 0.05), suggesting that the enhancement of fish growth is partly attributable to increases in PER by dietary methionine. Protein accretion is related to the metabolism of amino acids [32]. GOT and GPT are involved in protein and amino acids metabolism, and can be used to evaluate the utilization of essential amino acids in fish [55]. In the present study, the activities of GOT in the hepatopancreas and GPT in muscle were significantly improved by dietary methionine supplementation. However, the activities of GPT in the hepatopancreas and GOT in muscle were not affected. One possible explanation for this interesting result might involve alanine and aspartate, substance of GPT and GOT, respectively [56]. Studies have shown that dietary methionine had no effect on liver alanine content in rainbow trout [57], and muscle aspartate content in rat [58]. Accordingly, a lack of change in the concentrations of these substances might have been a partial contributor to the unchanged GPT in the hepatopancreas and GOT in the muscle of grass carp. However, the exact mechanisms of these changes await further characterization. Furthermore, hepatopancreatic GPT and muscle GOT activities of juvenile Jian carp were increased by MHA supplementation [59]. This difference in results between previous and present studies might be attributable to the different fish growth stages in the studies. In Atlantic salmon, dietary methionine increased protein retention in small fish but not in larger fish [54].

Fish growth is related to digestive and absorptive capacities, which in turn rely on the activities of digestive and brush border enzymes [9]. In the present study, the activities of intestinal amylase, trypsin, lipase, Na^+^/K^+^-ATPase, AKP and γ-GT decreased in fish fed the methionine-deficiency diet, and increased in fish fed optimal levels of methionine. Interestingly, the present results showed that, whereas optimal dietary methionine increased CK activity in the proximal intestine, CK activity in the mid intestine decreased, whereas there was no effect in the distal intestine. However, there is no information about the relationship between methionine and CK activity in vertebrates. Studies have shown that CK located near sites where ATP-dependent processes take place [60], and played a key role in the energy metabolism of cells with fluctuating energy requirements [61]. However, many nutrients, including essential amino acids, are absorbed via active transport which requires energy [62]. In Atlantic salmon, the absorption of methionine declined along the post-gastric intestinal tract [63]. This may explain, in part, the inconsistent effects of dietary methionine on CK activity in different intestinal segments in the present study. Additionally, there are several other possible reasons that the activities of digestive and brush border enzymes were improved by methionine in the present study. First, methionine is involved in the synthesis of spermine [64]. Spermine supplementation increased pancreatic enzyme activities in larval sea bass (*Dicentrarchus labrax*) [65]. Second, in rat, methionine enhanced the activity of Na^+^/K^+^-ATPase by maintaining the integrity of brain synaptosomes membrane [66]. Tissue integrity is positively associated with antioxidant defense of fish [67]. Methionine is a precursor of SAM, taurine, cysteine and spermine, which have been proved to be efficient antioxidants in terrestrial animals [64]. Thus, we next measured antioxidant status in the intestine and hepatopancreas of sub-adult grass carp.

MDA and PC are the most widely used biomarkers for oxidative damage of lipid and proteins, respectively [68]. In the present study, contents of MDA and PC were increased by methionine deficiency and decreased by optimal level of methionine in both the intestine and hepatopancreas of sub-adult grass carp. Lipid peroxidation and protein oxidation are mainly caused by attack of excessive ROS [68]. Superoxide anion and hydroxyl radical are two important oxygen free radicals that are strongly involved in oxidative damage [69]. In our study, superoxide anion-scavenging capacity and hydroxyl radical-scavenging capacity (measured as ASA and AHR activities, respectively) in the intestine, and hydroxyl radical-scavenging capacity (measured as AHR activity) in the hepatopancreas were improved by methionine supplementation, suggesting that methionine decreased oxidant damage potential partly via increasing the oxygen free radical scavenging capacity. Furthermore, in terrestrial animal, methionine and its intermediate metabolites (SAM and cysteine) can chelate metal ions such as Fe^2+^ and Cu^+^ [16, 17], and thus inhibit the formation of ROS [68]. Moreover, the methionine sulfoxide reductase system is a natural ROS scavenging system in yeast, bacteria and mice [15]. In zebrafish, methionine sulfoxide reductase is also expressed in the intestine [70]. Thus, the decreased oxidant damage in the presence of increased methionine might be partly related to the involvement of methionine in the chelation of metal ions and the methionine sulfoxide reductase system. However, this needs further experimental investigation in fish.

Oxygen free radical scavenging capacity largely depends on the antioxidant defense system in vertebrates. GSH is the major low-molecular-weight cellular antioxidant, and is capable of scavenging hydroxyl radical and singlet oxygen directly [68]. In the present study, GSH content in the intestine and hepatopancreas were increased by dietary methionine supplementation. Correlation analysis showed that ASA and AHR capacities were positively correlated with the intestinal GSH content (*r* = + 0.905, *P* < 0.05; *r* = + 0.915, *P* < 0.05), suggesting that increased GSH content due to methionine supplementation might contribute to enhanced capacities to scavenge superoxide anion and hydroxy radicals. Furthermore, increased GSH content due to methionine supplementation might be attributed to de novo synthesis of GSH and its regeneration from oxidized glutathione (GSSG). First, Methionine is an effective precursor of cysteine for GSH synthesis [71]. Second, GSH can be regenerated via reduction of GSSG by GR [68]. In the present study, methionine supplementation enhanced GR activities in both the intestine and hepatopancreas, and GSH content was positively correlated with GR activities in both organs (*r* = + 0.897, *P* < 0.05; *r* = + 0.967, *P <* 0.01).

In addition to low-molecular-weight antioxidants, antioxidant enzymes such as SOD, GPx, CAT and GST are also important defenses against ROS in fish [68]. In the present study, activities of SOD in the intestine and hepatopancreas, and GPx in the intestine were increased by methionine. Furthermore, ASA capacity was positively related to intestinal SOD activity (r = + 0.918, *P* < 0.01), showing that enhanced superoxide anion scavenging capacity might be partly due to increased SOD activity resulting from methionine supplementation. Interestingly, patterns of GST activities in the intestine and hepatopancreas, and CAT activity in the hepatopancreas were opposite to that of SOD. The reason for these interesting results is unclear. However, oxidant stress induced by methionine deficiency might explain this in part. In fish, CAT activity has been shown to increase following oxidative insults in the enterocytes of juvenile Jian carp [72] and in the liver of gilthead sea bream [19]. Furthermore, Bauchart-Thevret et al. [73] reported that methionine deficiency increased oxidant stress in the intestine of neonatal pigs. Thus, increased CAT activity might be related to an adaptive mechanism against stress. However, the exact underlying mechanism by which methionine influenced the antioxidant enzyme activities in fish is still unknown.

The present study showed that the quadratic regression model gave the least mean square error compared with the broken-line model, and was better fitted the relationship between methionine (or total sulfur amino acids) levels and the chosen responses. In the present study, the dietary total sulfur amino acids requirement of sub-adult grass carp (450-1, 170 g) based on the quadratic regression analysis for PWG was estimated to be 7.6 g/kg diet (27.2 g/kg protein) in the presence of 1.5 g cysteine/kg diet, which was lower than that reported for juvenile grass carp [25], juvenile Jian carp [2], juvenile Indian major carp (*Cirrhinus mrigala*) [74], juvenile *Labeo rohita* [75], juvenile Nile tilapia [76], and adult common carp [27] (Table 9). It is consistent with these studies that the total sulfur amino acids requirement for adult common carp [27] was lower than that for juvenile common carp [1, 26] (Table 9). Meanwhile, the optimal dietary protein level for very young salmonids is 45 to 50% of the diet, whereas juveniles require 40% and yearlings require about 35% dietary protein [62]. The metabolic rate of protein and amino acids decreased with increase of fish weight [62], thus this might contribute to the difference in total sulfur amino acids requirement for various growth stages of fish. Furthermore, the current results showed that dietary methionine deficiency could result in a decrease in digestive and absorptive ability, and antioxidant ability of the hepatopancreas and intestine in grass carp. It is quite necessary to evaluate the optimal methionine levels required for digestive and absorptive ability, and antioxidant ability of grass carp. Parameters of digestion, absorption and antioxidant capacity, such as activities of trypsin, Na^+^/K^+^-ATPase, glutathione peroxidase and anti-superoxide anion, and protein carbonyl content, have begun to be used as criteria for estimating the nutrient doses required for adequate function of fish digestive and antioxidant system respectively [77–80]. Based on the quadratic regression analysis for intestinal trypsin and hepatopancreatic AHR activities, dietary total sulfur amino acids requirements of sub-adult grass carp in the present study were estimated to be 8.5 g/kg diet (30.3 g/kg protein) and 6.9 g/kg diet (24.6 g/kg protein), respectively, in the presence of 1.50 g cysteine/kg diet, which were slightly different from that based on PWG in the present study. This might be attributable to the findings that the nutrient requirements of fish varied based on different physiological functions [67, 79].

**Table 9:** The comparison of dietary total sulfur amino acids (TSAA) requirement for fishes

Fish species	Weight, g	Diet CP, g/kg	Response variable	Estimated TSAA requirement	Reference
Grass carp (*Ctenopharyngodon idella*)	450-1, 170	280.6	Percent weight gain	7.6 g/kg diet (cysteine 1.5 g/kg diet) 27.2 g/kg protein	Results of the present study
			Intestinal trypsin	8.5 g/kg diet (cysteine 1.5 g/kg diet) 30.3 g/kg protein	
			Hepatopancreatic AHR	6.9 g/kg diet (cysteine 1.5 g/kg diet) 24.6 g/kg protein	
	12-40	370.0	Percent weight gain	12.1 g/kg diet (cysteine 1.1 g/kg diet) 32.7 g/kg protein	Wang [25]
Common carp (*Cyprinus carpio* L.)	550-1, 095	402.0	Final weight	12.8 g/kg diet (cysteine 4.2 g/kg diet) 31.8 g/kg protein	Schwarz et al. [27]
	2.35-3.87	385.0	Specific growth rate	28.1 g/kg diet (cysteine 20.0 g/kg diet) 73.0 g/kg protein	NRC [1] Nose [26]
Jian carp (*Cyprinus carpio* var. Jian)	9.9-29.8	350.0	Weight gain	15.0 g/kg diet (cysteine 3.0 g/kg diet) 42.9 g/kg protein	Tang et al. [2]
Indian major carp (*Cirrhinus mrigala*)	0.45-1.17	400.0	Weight gain	22.0 g/kg diet (cysteine 10.0 g/kg diet) 55.0 g/kg protein	Ahmed et al. [75]
*Labeo rohita*	2.4-8.1	400.0	Mean weight gain	12.9 g/kg diet (cysteine 1.4 g/kg diet) 32.3 g/kg protein	Murthy and Varghese [76]
Nile tilapia (*Oreochromis niloticus*)	1.28-9.16	280.0	Percent weight gain	8.5 g/kg diet (cysteine 0.4 g/kg diet) 30.4 g/kg protein	Nguyen and Davis [77]

## Conclusion

The present results demonstrated that methionine supplementation improved growth performance, enhanced digestive and absorptive function, and protected the hepatopancreas and intestine from lipid peroxidation and protein oxidation by improving enzymatic antioxidant capacity (SOD, GPx and GR activities) and non-enzymatic GSH content. Dietary methionine requirements for sub-adult grass carp (450-1, 170 g) based on PWG, intestinal trypsin and hepatopancreatic anti-hydroxy radical activities were estimated to be 6.12 g/kg diet (21.80 g/kg protein), 6.99 g/kg diet (24.90 g/kg protein) and 5.42 g/kg diet (19.31 g/kg protein) respectively in the presence of 1.50 g cysteine/kg (5.35 g/kg protein). Further study is needed to investigate the specific molecular mechanism by which methionine mediates antioxidant defense in fish.
